# Primary Headache in Yemen: Prevalence and Common Medications Used

**DOI:** 10.1155/2014/808126

**Published:** 2014-11-05

**Authors:** Salah A. Abdo, Mohammed Amood AL-Kamarany, Karem H. Alzoubi, Mohamed T. Al-Maktari, Abdulrhman H. Al-Baidani

**Affiliations:** ^1^Department of Pharmacy Practice, Faculty of Clinical Pharmacy, Hodeidah University, P.O. Box 3114, Hodeidah, Yemen; ^2^Tihama Foundation for Drug Studies and Research, Hodeidah, Yemen; ^3^Department of Clinical Pharmacy, Faculty of Pharmacy, Jordan University of Science and Technology, P.O. Box 3030, Irbid 22110, Jordan; ^4^Department of Medical Parasitology, Faculty of Medicine and Health Sciences, Sana'a University, P.O. Box 2289, Sana'a, Yemen

## Abstract

*Background and Objective*. Primary headaches is a major medical concern in certain Arabic countries, for example Oman, Jordan, and Qatar. This study was aimed at increasing understanding of the prevalence of headache in Arabic countries and identifying common medications used for treatment because of the lack of research done in this field in Yemen. *Methods*. This is a cross-sectional observational study conducted by recruiting case-series of adults and elderly who have primary headache within the age group from 18 to 85 years. 12640 subjects received a simple explanation for the aim of the study as ethical issue. The subjects were allowed to complete a self-conducted screening questionnaire. The data were diagnosed according to the International Headache Society's diagnostic criteria (2004). *Results*. The results showed that 76.5% of the primary headache is prevalent at least once per year, 27.1% of the tension type headache (TTH) was the maximum percentage of type of headache, and 14.48% of the migraine headache (MH) was the minimum percentage. On the other hand, the relationship between the primary headache and age of subjects was statistically significant (*P* < 0.05), while between primary headache and sex was not (*P* > 0.05). In addition, 70.15% of the subjects said that headache attacks affected their activity of daily livings (ADL). 62.26% of the subjects used the medications without medical advice regarding their headache. 37.73% of the subjects relied on medical professionals (physicians and pharmacist) regarding analgesics use. The most common agent used among the medications was paracetamol (38.4%). Others included ibuprofen, aspirin, diclofenac sodium, naproxen, mefenamic acid, ergotamine and (11.45%) were unknown agents. *Conclusion*. We concluded that absence of health attention from the Yemeni Community and education from the health system in the country regarding analgesics use and their potential risk led to abuse of such medications and could be a reason beyond high prevalence of headache in Yemen.

## 1. Introduction

Headache can be defined as prevalent and disabling condition affecting people in all age groups worldwide, resulting in low job performance and quality of life with a significant economic burden on societies [1]. Headache gains attention worldwide because it is a common discomfort making to the top ten list of complaints in ambulatory medical care [2] and has a low healthcare and public profile [3]. Moreover primary headaches rank among the most common and disabling disorders worldwide [4].

The impact of headache is incredible, for example, in children, one of the causes that result in absence from school and interfering with other daily activities [5, 6]. On the other hand, in elders the incidence of most primary headache may decline after 55–60 years of age [7, 8]. The prevalence of primary headache in certain studies in adults over 18 years accounts for about 6.4% of the population [9, 10]. If we go further toward Asia, one study mentioned that the prevalence of migraine is between 1 and 22% and that is lower than that reported for North America and Europe [11]. Furthermore, there is a lack in epidemiology of headache disorders in Asia [11]. In addition, there is an overuse for analgesics and that may be the cause beyond the high prevalence in certain Middle East areas such as Oman, Qatar, and Jordan where there is high prevalent self-medicating for headache [12, 13] because the overuse of such analgesics can lead to overuse syndrome [14, 15].

Several studies of prevalence of primary headaches in Arabic countries were conducted, for example, Saudi Arabia, Qatar, Oman, Bahrain, United Arab Emirates, Kuwait, and Jordan [2]. The major objective of our study is to help in understanding of distribution of primary headaches in Arab area and correlate with age and gender. In addition, to estimate the common medications used by Yemeni people.

## 2. Materials and Methods

### 2.1. Study Design

This is a cross-sectional observational study conducted by recruiting case-series of adults and elderly who have primary headache within the age group from 18 to 85 years. Subjects with primary headache received a simple explanation for the aim of the study as an ethical issue. If they agreed, the subject was interviewed. Confidentiality of the collected data was achieved by keeping data record in a locked room with limited access to the research team only.

### 2.2. Subjects and Questionnaire

The study included 12640 subjects from four Yemeni governorates that were selected to estimate the one-year overall prevalence in Yemen; they include Sana'a, Taiz, Al-Hodeidah, and Thamar. Subjects were approached at their work places, classes, or homes and selected in random manner. The study was conducted from July 2010 to September 2011. The study was carried out by using the principles described in the Declaration of Helsinki, including all amendments and revisions. Every subject was asked to complete a self-conducted questionnaire in the presence of the researcher to answer any inquiries and then reviewed immediately to detect and prevent any errors. Researchers interviewed illiterate subjects to complete the questionnaire. The questionnaire gathered information that included demographic data, family history, frequency and type of headache, and its impact on everyday activities, medications use, consultation regarding medication increasing with time, and frequency of headache after medication usage. The data were diagnosed according to the International Headache Society's diagnostic criteria [16].

### 2.3. Data Analysis

Statistical Package for the Social Sciences version 16.0 (SPSS Inc., Chicago, IL, USA) and Excel software version 2010 were used for data analysis. The descriptive analysis (%) and Chi-square test were used to make comparisons among categorical variables. For all statistical analyses, a *P* value of less than 0.05 was considered statistically significant.

## 3. Results

### 3.1. Demographic Characteristics of Subjects

The sample was of subjects (*n* = 12640) that were selected from four different governorates of Yemen within the age group from 18 to 85 years. The sample of study was represented in the males 67.7% while in the females it was 32.3%. As shown in Table 1, 33.8% of the subjects were high school graduates followed by bachelor degree holders (19%), diploma holders (16.24%), university students (8.98%), secondary school graduates (7.7%), master's degree holders (5.9%), and Ph.D. degree holders (0.56%). 98.8% were Yemeni people. 57.6% were singles and 40.2% were married. 23.3% were smokers and 76.7% were nonsmokers.

### 3.2. Prevalence of Primary Headache

The primary headache was found in 76.6% (*n* = 9684); the characteristics of patients were shown in Table 2. Two types of primary headache were observed in this study, tension type-headache (TTH) was 27.10%, and migraine headache (MH) was 14.50%, while unknown type was 35.01%. However, this difference between types of headache was statistically significant (*P* < 0.05). On the other hand, the relationship between the primary headache and age of subjects was found and was statistically significant (*P* < 0.05), while the relationship between primary headache and sex was not (*P* > 0.05) (Table 3). In addition, the higher frequency of TTH and MH was in adults between 18 and 29 years (76.85% and 70.30%, resp.), and the lower frequency was in elderly more than 50 years (4.20% and 4.40%, resp.). Also, 70.15% of the subjects said that headache affected their activity of daily livings (ADL), and 64.1% had positive family history of headache.

### 3.3. Common Medication Used

**Figure 1:**
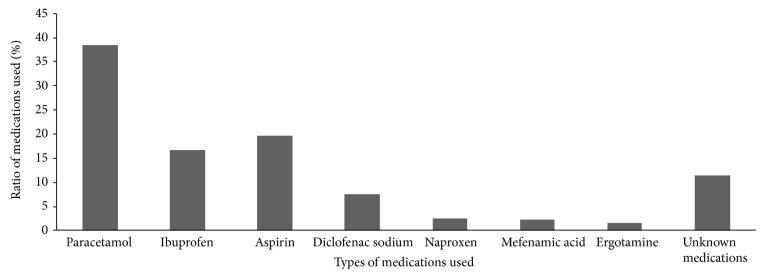
Medications use among headache patients (*n* = 10996). Note: some patients use different medications at an increase of headache frequency.

The use, abuse, and incidence of medications in 9684 patients with primary headache were studied. The common medications used for headache were paracetamol (38.4%), ibuprofen (16.7%), aspirin (19.7%), diclofenac sodium (7.5%), naproxen (2.5%), mefenamic acid (2.2%), ergotamine (1.5%), and unknown (11.5) (Figure 1). In other meaning, 88.5% of medications were known by their users, while 11.5% were unknown. Furthermore, majority of subjects (62.26%) used the medications without medical advice and few of them did (37.73%). In addition 64.8% of subjects depend on family to get medications while 35.16% depend on physicians and pharmacists. 8.75% of patients used analgesics on daily basis, fewer than daily to weekly was 24.58%, fewer than weekly to monthly was 27.90%, fewer than monthly to one year was 24.79%, and patients who never use analgesics was 13.90%. In addition, 77.57% of subjects showed increased headache frequency on medication use, while 22.42% did not** (** Table 4).

## 4. Discussion

The overall one year period prevalence of primary headaches among adults in Yemen was estimated for the first time. About 76.6% of subjects complained from primary headache at least once per year. Primary headache included two types, migraine and tension-type headache. The prevalence in this study was much higher comparing with average global prevalence of headache (46%) [11, 17]. Similar results were reported in Arabic countries, namely, Oman and Qatar [18, 19]. Also, the same results were recorded in non-Arabic countries, namely, Northern Finland and Singapore [20–23]. In addition, previous study was reported in Oman that showed a prevalence of headache of about 45% [24]; however, that study was among university medical students and not the general population. Other studies were performed in Arabic countries that showed high prevalence, except Saudi Arabia in which the prevalence of primary headache is ranged from 8 to 12% [19, 25, 26], which was much lower than that reported in all other studies from the Middle East area, while in Jordan the prevalence of primary headache was also higher than overall global prevalence [2].

On the other hand, Table 5 showed that TTH was more prevalent than that of MH (27.10% and 14.5%, resp.) and it was within the prevalence range reported from studies conducted in the Middle East area (3.1–36.1%) [3, 18, 24, 27, 28]. Furthermore, our study was in concordance with other studies in Middle East area that showed that prevalence for tension-type headache was lower than its global prevalence (about 42%) [11], which seems to be the case for the whole Middle East area. Concerning MH, the prevalence in our study was higher than that showed in Arabic countries which was falls within 10 to 12.2% [18, 28, 29]. One study in Qatar showed that the prevalence was 7.9 %. In our study, the prevalence of MH was higher than the global MH prevalence (11.2%) [17], and in Western Europe (14%) [18] which was very close to that in our study [17]. Therefore, Yemen seems to have higher migraine prevalence than many other parts of the world.

In agreement with Alzoubi et al. [2], the family history of headaches was found in most of the subjects. Furthermore, ADL was affected more significantly than in that study, 70.1% and 51.6%, respectively. About 62.26% of our sample did not seek medical advice regarding their headaches.

The most common medication used in Yemen was paracetamol. Same result obtained from Jordan, where the only study discussed this issue. In addition most users in Jordan, rely on recommendations from non—medical advisers (the family members), this in agreement with Alzoubi et al with exception that usually users rely on friends rather than family members but both indicating a lack of communication with health professionals, physicians and pharmacists. In the same study the causes beyond popularity of paracetamol could be due to its availability as an over-the-counter medication, low price, and its safety and less side effects profile on the gastrointestinal tract [2].

The percentage of non-smokers was 76.7 % and smokers was 23.3% that was lower than in Jordan and surrounding countries reported smoking prevalence of about 26–48% [30–33]. In agreement, our prevalence of smoking falls within the overall prevalence of smoking among adult males and females (21 to 37%) in the high-income countries and low to middle income countries (8.9 to 49%) [34]. Also, in agreement with other studies conducted in Arabic area the prevalence of headache is more in female than in male and the prevalence tended to be higher in younger patients below the age of 40 [19, 20].

## 5. Conclusion

In conclusion, 76.5% of our subjects have headache attacks at least once per year indicating that headache that is a major health problem in Yemen. In addition, young adults were the most affected especially by TTH. Absence of health attention and education regarding analgesic use led to abuse of such medications and could be one of the reasons beyond development of headache and this is why Arabic countries have the greatest prevalence of headaches. On the other hand, the educational programs should be planned and implemented to ensure safe practices and to limit random usage of analgesics and encourage population to seek for medical advice before administration.

## Figures and Tables

**Table 1:** Demographic data (*n* = 12640).

Characteristic	*n*	%
Gender		
Male	8562	67.7
Female	4078	32.26
Age (years)		
18–29	8754	69.25
30–39	2404	19.01
40–49	962	7.61
≥50	520	4.11
Education		
Illiterate	570	4.5
Elementary school	394	3.11
Secondary school	975	7.71
High school	4281	33.86
Diploma	2053	16.2
University student	1136	8.98
B.A.	2407	19.04
M.S.	752	5.94
Ph.D.	72	0.56
Nationality		
Yemeni	12492	98.8
Arab	146	1.15
Foreign	2	0.016
Marital status		
Single	7283	57.6
Married	5084	40.2
Other	273	2.2
Monthly income		
Low (<232$ US)	9318	73.7
Medium (232–462$ US)	2594	20.5
High (>462$ US)	728	5.7
Smoking		
Smoker	2945	23.3
Nonsmoker	9695	76.7

**Table 2:** Family history, frequency and type of headache.

Variable	*n*	%
Headache frequency (*n* = 12640)		
Daily	1087	8.6
Fewer than daily to weekly	2737	21.6
Fewer than weekly to monthly	2998	23.7
Fewer than monthly to 1 year	2862	22.6
No headache	2956	23.4
Headache affects daily activities (*n* = 9684)		
Yes	7286	70.2
No	2398	29.8
Other family members complaining from headaches (*n* = 12640)		
Father	1006	7.9
Mother	1956	15.50
Brothers or sisters	1527	12.10
Other relatives	2342	18.50
More than one family member	1269	10.39
None	1584	12.50
No headache	2956	23.40
Type of headache (*n* = 12640)		
Migraine	1831	14.50
Tension	3427	27.10
Unknown	4426	35.01
No headache	2956	23.40

**Table 3:** Prevalence of migraine and tension-type headache according to ages and gender (*n* = 5258).

Type	TTH							MH						
Gender	Male		Female		Total		*P* value	Male		Female		Total		*P* value
Age	*n*	%	*n*	%	*n*	%		*n*	%	*n*	%	*n*	%	
18–29	1278	37.3	1434	41.8	2712	76.85	*P* < 0.05^*^	641	35.1	644	35.2	1285	70.30	*P* < 0.05^*^
30–39	226	6.60	174	5.10	400	11.70		191	10.4	137	7.5	328	17.90	
40–49	52	1.50	119	3.50	171	5.0		78	4.3	59	3.2	137	7.50	
≥50	59	1.70	85	2.50	144	4.2		53	2.9	28	1.5	81	4.40	
Total	**1615**	**47.10**	**1812**	**52.90**	**3427**	**100**		**963**	**52.7**	**868**	**47.4**	**1831**	**100**	

*P* value	*P* > 0.05							*P* > 0.05						

^*^The relationship between the primary headache and age of subjects was statistically significant (*P* < 0.05), while the relationship with sex was not statistically significant (*P* > 0.05). TTH: tension-type headache; MH: migraine headache.

**Table 4:** Approach to medication use among headache patients.

Variable	*n*	%
Nonadvice medical help for headaches (*n* = 9684)		
Nonadvice	6030	62.26
Advice	3654	37.73
Advice on using analgesics (*n* = 3654)		
Physician	993	27.71
Pharmacist	2661	72.82
Medications usage (*n* = 9684)		
Daily	848	8.75
Fewer than daily to weekly	2381	24.58
Fewer than weekly to monthly	2702	27.90
Fewer than monthly to 1 year	2401	24.79
No use	1347	13.90
Increase in headache frequency after analgesic use		
Yes	2172	22.42
No	7512	77.57
Increase analgesic dose used over time		
Yes	3335	34.34
No	6349	65.56

**Table 5:** Prevalence of TTH and MH in Arab countries.

Country	Prevalence of Headache (%)	TTH (%)	MH (%)
Our study^a^	76	27.10	14.50
Jordan^b^	82.3	36.1	7.7
Oman^c^	83.6	—	10.1
Qatar^d^	72.5	11.2	7.9
Saudi Arabia^e^	8–12	3.1–9.5	2.6–5

TTH, tension type headache; MH, migraine headache.

^a^Adult population (≥18 years old) for 24-month prevalence.

^b^Adult population (≥18 years old) for 24-month prevalence.

^c^Population (>10 years old) for 24-month prevalence.

^d^Adult population (>15 years old) for 3-month prevalence.

^e^All population for 6-month prevalence.
